# Clinical Utility of Belay Summit™ Cerebrospinal Fluid Test to Inform Diagnosis and Management of Central Nervous System Cancer—A Single Institution Case Series

**DOI:** 10.3390/cancers18071094

**Published:** 2026-03-27

**Authors:** Michael Youssef, Alexandra Larson, Vindhya Udhane, Zhixin Jiang, Daniel Lim, Jennifer N. Adams, Rakshitha Jagadish, Anthony Acevedo, Brett A. Domagala, Samantha A. Vo, Tarin Peltier, Daniel Sanchez, Viriya Keo, Julianna Ernst, Kala F. Schilter, Qian Nie, Honey V. Reddi

**Affiliations:** 1 Department of Neurology and the Department of Hematology and Oncology at UT Southwestern Medical Center, Dallas, TX 75235, USA; michael.youssef@utsouthwestern.edu (M.Y.);; 2 Belay Diagnostics, 1375 W. Fulton St, Suite 530, Chicago, IL 60607, USA; alarson@belaydiagnostics.com (A.L.);

**Keywords:** CSF liquid biopsy, summit test, clinical utility, genomic alterations, CNS tumors

## Abstract

Background: Cytology from cerebrospinal fluid (CSF) is standard-of-care to detect central nervous system (CNS) cancers but suffers from low-sensitivity and lacks associated molecular information, often requiring brain biopsy or resection to obtain. Belay Diagnostics offers analytically and clinically validated CSF-based tests to support the diagnosis and management of primary and secondary CNS cancers. However, the clinical utility (CU) of these assays has not been previously evaluated. Methods: This retrospective study presents a real-world, single institution experience of using the Belay Summit liquid biopsy test for all orders received (*n* = 123) between October 2024 and September 2025. Clinical information was reviewed for demographics, provisional diagnosis, oncology history, CSF cytology results, and tumor genomic profiling results. The primary endpoint of this study was to evaluate the impact of Belay CSF-based assays on physician diagnosis and treatment decisions. Secondary endpoints included evaluating the clinical performance of the Belay Summit test verses cytology in CNS malignancy detection (sensitivity, specificity, and accuracy). Results: The cohort included 120 patients with suspected or previously diagnosed primary (*n* = 40) or metastatic (*n* = 80) CNS tumors; three patients completed longitudinal testing for a total of 123 specimens and test orders. Summit showed higher sensitivity for CNS malignancy (90%) over CSF cytology (12%). The Belay CSF liquid biopsy test demonstrated strong clinical utility and was essential to clinical course pursued in 93% (114/123) of specimens, informing treatment and management decisions. Conclusions: The Belay Summit test provides clinically meaningful information to support physician decision-making for the diagnosis and management of primary and secondary CNS tumors, especially in cases where tissue sampling is infeasible.

## Simple Summary

Detecting central nervous system (CNS) cancers from evaluation of cells in cerebrospinal fluid (CSF) has very low positivity rates, limiting downstream management of these cancers. The Belay Summit CSF-based test has been shown to accurately indicate the presence of cancer in 90% of cases. This study presents the results from a single institution using the Belay Summit liquid biopsy test showing higher sensitivity over CSF cytology testing, providing clinically meaningful information to support physician decision-making for the diagnosis and management of CNS cancers.

## 1. Key Points

- The Belay Summit cerebrospinal fluid liquid biopsy test demonstrated clinical utility to inform diagnosis and subsequent management of patients with primary and secondary central nervous system tumors.
- The Belay test demonstrates higher sensitivity and specificity in the detection of leptomeningeal disease when compared to cerebrospinal fluid cytology.

## 2. Importance of the Study

This single-institution case series highlights the clinical utility (CU) of the Belay Summit cerebrospinal fluid (CSF)-based liquid biopsy test to aid in diagnosis and management of patients with suspected or confirmed primary and secondary central nervous system (CNS) tumors. The CU of this test is particularly evident in cases where biopsy or resection is infeasible, including suspected leptomeningeal disease (LMD) and deep-seated tumors.

## 3. Introduction

CNS cancers are a varied group of neoplasms that present unique challenges in terms of diagnosis, treatment, and monitoring. They are categorized into two main types, primary tumors, which originate in the CNS, and secondary (i.e., metastatic) tumors, which spread to the CNS from other parts of the body [1]. Each year, more than 200,000 people in the United States develop parenchymal metastases in brain and spinal cord tissue and 110,000 people are diagnosed with leptomeningeal metastases in membranes surrounding the CNS [2,3]. Notably, LMD occurs in about 5–20% of systemic cancer patients and is associated with particularly poor prognoses [4]. Despite improvements in surgery, radiation, and chemotherapy, outcomes for brain tumors are still dismal and pose substantial therapeutic challenges with limited curative options [5].

Traditional diagnostic methods like neuroimaging and CSF cytology, while foundational, often exhibit low-sensitivity and specificity in the diagnosis of brain malignancies and LMD [6,7,8]. The National Comprehensive Cancer Network (NCCN) Guidelines now recommend the use of molecular testing to aid in CNS tumor diagnostic work-up [9]. However, sampling tumor material can be a challenge due to the inherent risks of neurosurgical biopsy and the associated morbidity and mortality [8]. Furthermore, the absence of tumor molecular data can make it especially difficult to distinguish treatment effects from tumor progression [6,8]. Recent advances in molecular diagnostics have led to the development of CSF-based liquid biopsy assays that can detect genomic changes in tumor-derived DNA found in CSF (CSF-tDNA). This minimally invasive test has the potential to offer earlier and more accurate diagnoses, real-time disease monitoring, and personalized treatment planning for patients with CNS tumors [10]. In contrast to plasma-based liquid biopsies that face sensitivity challenges posed by the blood–brain barrier (BBB) [11], CSF offers a more reliable medium for detecting tumor-derived genomic alterations within the CNS compartment [12,13].

Among the novel diagnostic tools emerging in this space is the Belay Summit test that evaluates CSF-tDNA simultaneously for genomic (gene-level and chromosomal arm-level alterations) and epigenomic (*MGMT* promoter methylation) molecular markers [14,15] from low input DNA. Summit is a clinically validated assay that leverages proprietary MethySaferSeqS duplex sequencing technology [16] to analyze CSF-tDNA and detect key genomic alterations and chromosomal abnormalities with 90% sensitivity and 95% specificity [14]. This test marks a significant advancement in the molecular characterization of both primary CNS tumors and secondary metastatic involvement of the CNS, particularly in cases where conventional diagnostics are inconclusive.

In this study, we present the CU of the Belay Summit CSF-based liquid biopsy test in a retrospective case series cohort of all patients referred to Belay from a single institution between October 2024 and September 2025. Clinical information that was provided with the test orders was evaluated in the context of Summit results and clinical outcomes as determined by the ordering physician to demonstrate CU. Test results were compared to CSF cytology to evaluate relative sensitivity and highlight cases in which liquid biopsy provided critical diagnostic insight that impacted clinical management.

## 4. Materials and Methods

### Clinical Cohort and Retrospective Analysis

This study evaluated data from CSF specimens that were submitted to Belay Diagnostics, Chicago, USA for clinical testing via Summit [14] with or without *MGMT* promoter methylation [15] testing from a single institution. A total of 123 CSF samples were received between October 2024 and September 2025 from 120 unique patients, three of whom each contributed two samples for longitudinal analysis. The Summit test interrogates CSF-tDNA for mutations in 32 genes (single nucleotide variants (SNVs), multi-nucleotide variants (MNVs) and insertions/deletions (indels)) along with chromosome arm-level aneuploidy [14] and optional analysis of *MGMT* promoter methylation [15]. Summit is clinically validated to detect CSF-tDNA with a sensitivity of 90% and specificity of 95% with 0.3% variant allelic frequency (VAF) limit of detection [14].

Clinical information provided for testing was reviewed for demographics (age in years, sex assigned at birth, race), location of the lesion, and CSF collection site (lumbar puncture (LP), or Ommaya reservoir) as well as the provisional diagnosis and available parallel diagnostic work-up including, when available, imaging, pathology, CSF cytology results, and tumor genomic profiling results. CU outcomes were determined by considering Belay test results in the context of broader diagnostic findings and subsequent changes in care management. Namely, post-Belay testing, cases were reviewed by the ordering provider physician for whether Summit-informed CNS malignancy diagnosis, treatment selection (chemotherapy, radiation therapy or targeted therapy), or management decisions (transition to hospice, observation, or specialist referral) or confirmed the absence of CNS malignancy or therapeutic response.

## 5. Results

### 5.1. Cohort Demographics and Clinical Pathology

The cohort is composed of 41 males and 79 females, with the majority of individuals (*n* = 61; 51%) being of white ethnicity (Table 1). Most individuals were in the >65 years age group (*n* = 43, 36%) with the 21–50 years and 51–65 years age groups each accounting for 28% (*n* = 34) and 33% (*n* = 40) of individuals, respectively. For the 120 patients, the provisional diagnosis prior to Belay testing included suspected primary CNS disease (*n* = 40, 33%) and suspected metastatic disease with or without concerns for leptomeningeal involvement (*n* = 80, 67%). Summit was ordered independently in 51% of cases (*n* = 63) and included *MGMT* promoter methylation status evaluation in 49% of cases (*n* = 60).

The majority of the CSF specimens (114 of 123, 93%) were collected via LP as opposed to an Ommaya reservoir (Table 2). Confirmed or suspected disease prompting Belay testing was most often located in the brain (*n* = 94, 76%) rather than the spine (*n* = 8, 7%), with the remainder in both locations (Table 2). The most commonly listed symptoms were cranial nerve deficits (*n* = 37, 30%) followed by headaches (*n* = 35, 28%), no symptoms reported (*n* = 27, 22%), and motor difficulties (*n* = 27, 22%).

### 5.2. Positivity Rate of Summit

**Figure 1:**
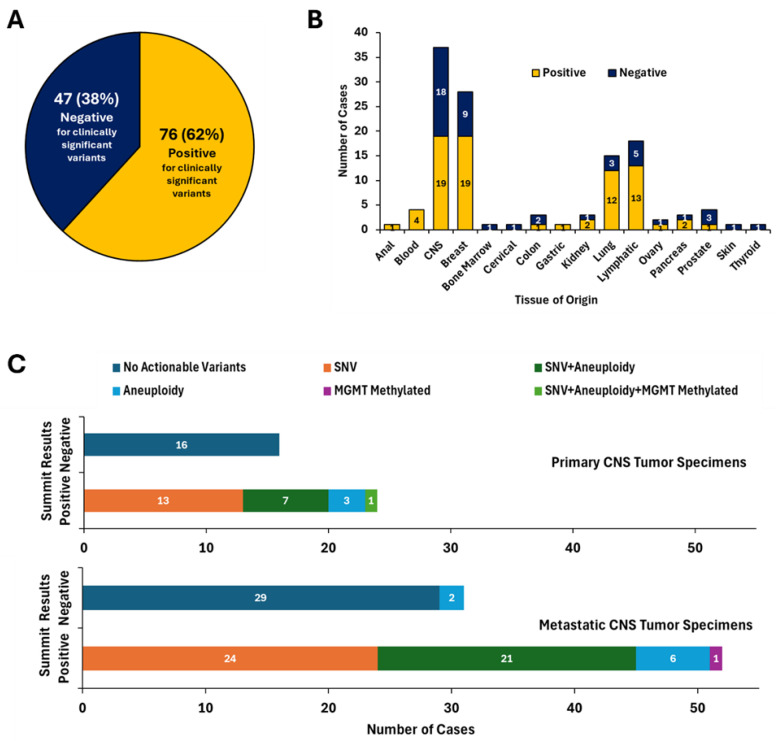
Overview of Summit results for 123 cerebrospinal fluid (CSF) specimens. Summit detected a clinically significant variant in 62% of specimens (yellow); 38% of samples were negative for clinically significant variants (navy blue) (**A**). Distribution of positive and negative Summit results in suspected primary or metastatic central nervous system (CNS) disease of varying tissues of origin (**B**). Clinically actionable single nucleotide variants (SNV) were detected with or without chromosomal arm-level aneuploidy and *MGMT* promoter methylation in suspected primary and metastatic CNS specimens (**C**).

**Figure 2:**
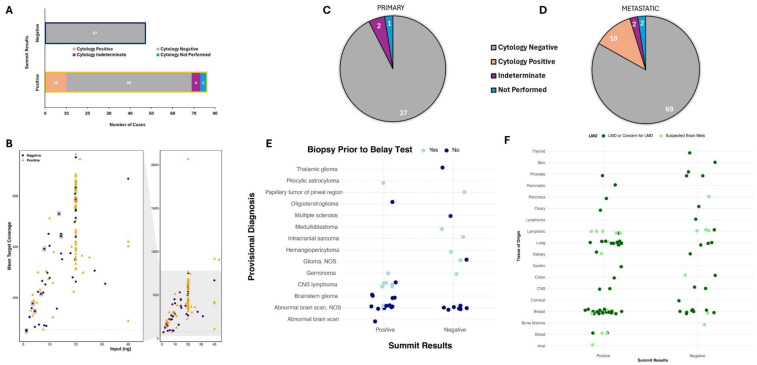
Summit demonstrated higher sensitivity compared to cerebrospinal fluid (CSF) cytology (**A**), which was not impacted by DNA input (**B**). The majority of suspected primary and metastatic tumors had negative CSF cytology results at the time of Belay testing (**C**,**D**). Summit positivity in primary central nervous system (CNS) tumors in the context of biopsy prior to Belay test (**E**) and, Summit positivity in metastatic CNS tumors in the context of clinical suspicion of LMD or suspected brain metastasis (**F**). Red boxes indicate false negative specimens and the green box indicates the false positive specimen. Not otherwise specified, NOS; leptomeningeal disease, LMD; metastasis, mets.

Of the 123 specimens submitted for Summit, over half (*n* = 76, 62%) were reported as positive (Figure 1A,B). Positive specimens were found to have at least one variant with clinical actionability as defined by AMP/ASCO/CAP guidelines [17], often including key biomarkers defined by the WHO [1] and NCCN [9] guidelines and/or a high level of aneuploidy (>5 arm-level gains or losses). Variants of clinical significance were detected in 60% (24 of 40) of cases with concern for primary CNS cancer and 63% (52 of 83) of metastatic cases (Figure 1C). Of the 123 samples, 120 had prior or parallel CSF cytology completed at the time specimens were sent for Belay testing with a significant majority of the cohort, 86% (*n* = 106) being negative for malignant cells (Figure 2A). About 8% (*n* = 10) showed cytological evidence of malignancy, and 3% (*n* = 4) were indeterminate. The 10 CSF cytology positive samples had clinically significant variant(s) detected by Summit, demonstrating 100% concordance between Summit and CSF cytology when the CSF cytology was positive. Summit was positive in 59 of the 106 (56%) CSF cytology negative cases and 4 were indeterminate (100%) CSF cytology cases. All 47 Summit-negative cases were negative by CSF cytology (Figure 2A). There did not appear to be a correlation between DNA input (ng) or mean gene-specific primer (GSP) target coverage and Summit positivity or negativity (Figure 2B).

Evaluation of the primary versus metastatic cohorts showed that 37 of the 40 primary and 69 of the 83 metastatic specimens received for Summit testing were negative for CSF cytology (Figure 2C,D). In the primary CNS cohort, patients in 15 of the 40 (38%) cases had undergone biopsy prior to sending for Belay testing (Figure 2E). The majority of cases did not have a prior biopsy completed and concern for malignancy was based on abnormal findings on MRI. Among the 83 metastatic specimens, a prior concern for or confirmation of LMD was noted in 64 (77%) specimens with the underlying primary tissue malignancies including breast (*n* = 28), lung (*n* = 15), lymphatic (*n* = 11), central nervous system (*n* = 4), blood (*n* = 4), prostate (*n* = 4), pancreas (*n* = 3), kidney (*n* = 3), colon (*n* = 3), ovary (*n* = 2), skin (*n* = 1), bone marrow (*n* = 1), thyroid (*n* = 1), anal (*n* = 1), cervical (*n* = 1), and gastric (*n* = 1) (Figure 2F). Of the 64 cases with concern for or prior confirmation of LMD, Summit detected clinically significant variants in 40 (63%) samples.

### 5.3. Genomic Alterations Detected by Summit

**Figure 3:**
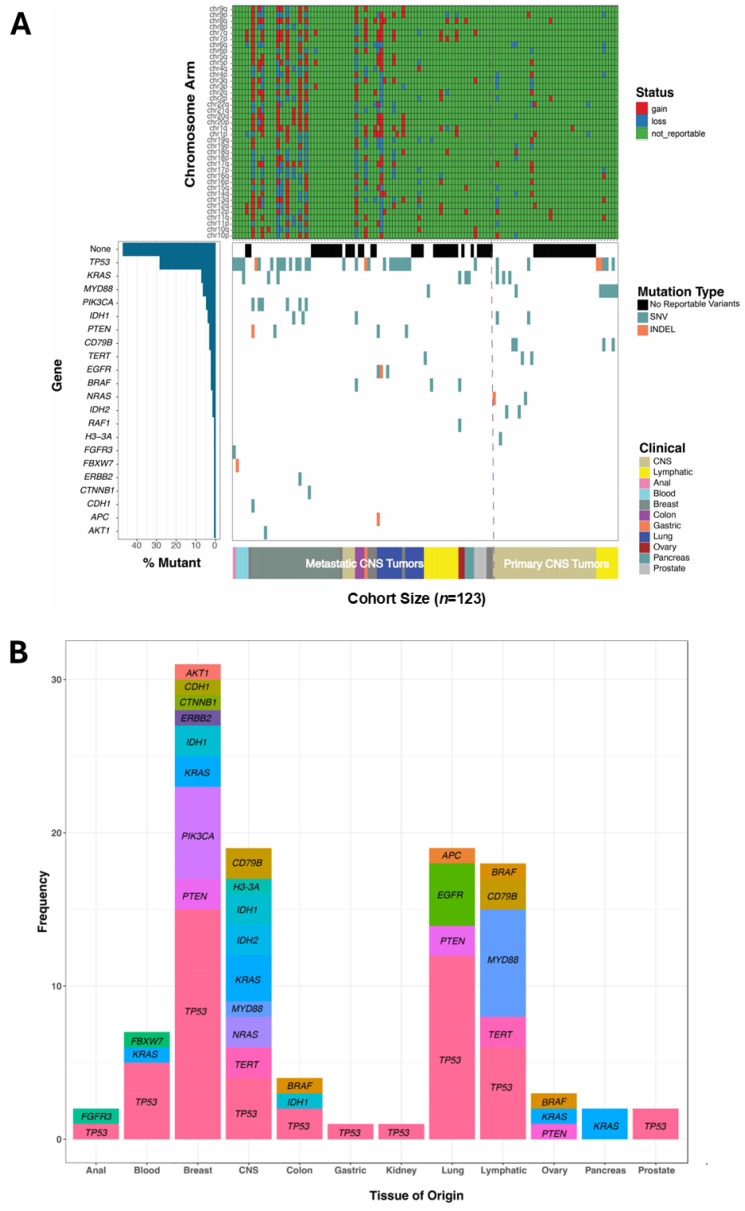
Summary of Summit results for 123 cerebrospinal fluid (CSF) samples. Summit detected gene-level variants and chromosomal arm-level alterations (**A**). Clinically significant variants were detected in 21 of the 32 genes included on the Summit panel with different frequencies between tissues of origin (**B**). SNV, single nucleotide variant; INDEL, insertion/deletion; central nervous system, CNS.

Summit detected clinically significant variants in 24 of the 40 (60%) CNS specimens for which a primary malignancy was suspected and in 52 of 83 (63%) metastatic specimens (Figure 3A). Chromosome arm-level aneuploidy was detected in 11 (28%) primary and 28 (34%) metastatic Summit positive specimens. Across the 123 specimens, gene-level variants were detected in 66 (54%) cases with variants being identified in 21 of the 32 genes included on the Summit panel (Figure 3).

In both primary and metastatic specimens, the most commonly altered gene was *TP53* with 42 different SNVs and Indels seen across 35 specimens followed by *KRAS* with alterations in nine specimens (Figure 3B). Variants in several genes, namely *H3-3A*, *NRAS*, *IDH2*, and *CD79B*, were only detected in primary CNS disease specimens. In contrast, variants in *CTNNB1*, *CDH1*, *AKT1*, *PIK3CA*, *PTEN*, *EGFR*, *BRAF*, and *RAF1* were found only in metastatic cancer specimens. When aneuploidy was observed, chromosomal arm-level gains and losses were detected for multiple chromosomes, with the highest levels of aneuploidy (gains or losses of every or nearly every chromosome) being observed in metastatic breast and lung specimens (Figure 3A). Only four cases of isolated aneuploidy events were observed: chr10q gain in metastatic breast carcinoma, chr6q loss in a case with primary central nervous system lymphoma, chr1p gain in metastatic small-cell lung carcinoma, and chr19q loss in metastatic diffuse large B-cell lymphoma. Gain of chr17q12, containing *ERBB2*, was reported for three patients; two had a history of breast cancer and one a history of lung cancer. Gain of chr7p11.2, containing *EGFR*, was reported for six patients with histories of gastric, blood, lymphatic, colon, and breast primaries.

Prior tumor and/or liquid biopsy genomic profiling testing completed by other laboratories was available for 17 of the 83 metastatic cases and reviewed for concordance with Summit results (Table 3). Most testing had been performed when the primary cancer was diagnosed, often over a year prior to concern for CNS metastasis and Belay testing. Genomic findings varied between tumor, plasma, and CSF (Summit) in multiple cases. In 10 of the 17 samples (59%), Summit detected some or all variants previously found in the primary tumor or plasma.

### 5.4. Variant Allelic Frequency of Gene Variants Detected in Leptomeningeal Disease

**Figure 4:**
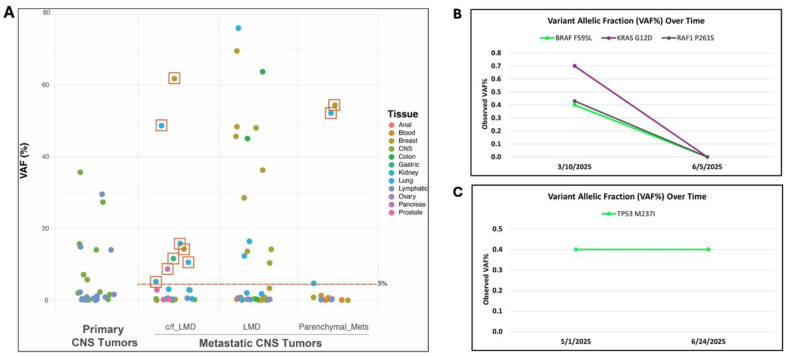
Variant allelic frequency (VAF) can be used as a quantifiable measure to inform diagnosis of leptomeningeal disease (LMD) and track disease progression and/or response to therapy. (**A**) VAF greater than 5% reliably informed diagnosis of LMD). Red squares indicate orders with initial concern for (c/f) parenchymal metastasis (mets) and/or leptomeningeal involvement that were confirmed to have LMD upon neuroimaging/clinical presentation after Belay testing, correlating with VAF of detected variants. VAF of clinically significant variants detected in two longitudinally collected specimens was used to monitor disease progression and therapeutic response (**B**) or resistance (**C**). central nervous system, CNS.

Clinical follow-up of Summit positive cases with initial concern for leptomeningeal involvement confirmed that samples with >5% VAF were found to have LMD. A higher VAF (>5%) was observed in cases with known LMD compared to those with suspected parenchymal metastatic tumors (Figure 4A). Further diagnostic work-up following Belay testing confirmed that the two cases with initially suspected parenchymal metastasis and VAF >5% were found to also have LMD. In a case of LMD from metastatic ovarian cancer, longitudinal testing through Belay (study IDs 30 and 31, Table 4) confirmed therapeutic response when previously identified variants were not detected following several weeks of intrathecal (IT) methotrexate (Figure 4B). These findings aligned with stable leptomeningeal enhancement on MRI as well as patient symptomatic improvement and clinical stabilization. In contrast, longitudinal testing (study IDs 23 and 66, Table 4 and Table 5) of a metastatic breast cancer with LMD detected the same *TP53* variant at the same VAF after treatment with IT chemotherapy (Figure 4C). The most recent MRI for this patient, completed a week prior to specimen collection, demonstrated a slight decrease in leptomeningeal enhancement compared to prior scans.

### 5.5. Clinical Utility of Belay Testing to Aid in Diagnosis and Management

**Figure 5:**
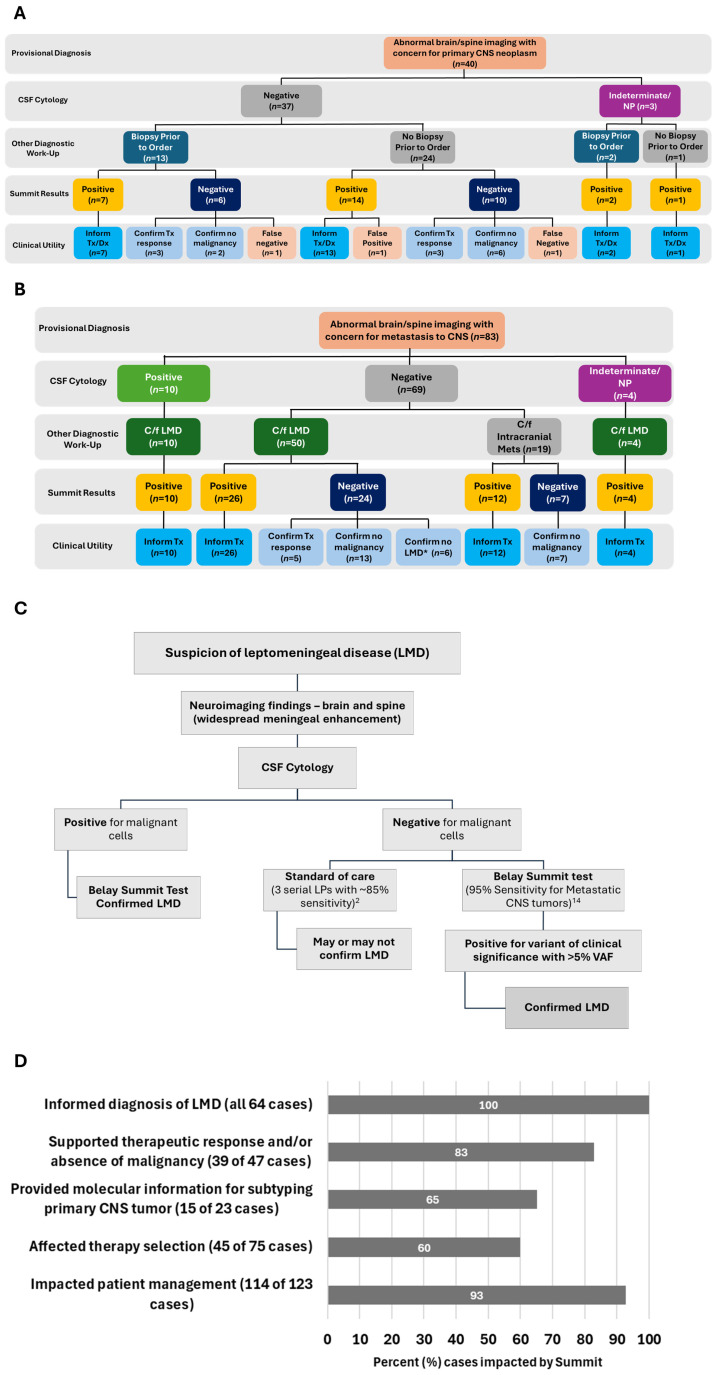
Summit impacts treatment and management of primary and secondary central nervous system (CNS) malignancies. Graphical representation of the clinical utility (CU) of Summit alongside cerebrospinal fluid (CSF) cytology following a provisional diagnosis of primary (**A**) or metastatic (**B**) CNS disease for 123 samples. Schematic of testing algorithm implemented at University of Texas, Southwestern to inform diagnosis of leptomeningeal disease (LMD) (**C**). Breakdown of Summit CU impact among 123 samples (**D**). Not performed, NP; treatment, Tx; diagnosis, Dx; cerebrospinal fluid, CSF; lumbar puncture, LP; variant allelic frequency, VAF.

In the primary CNS cancer cohort, positive Summit results informed diagnosis and/or management in 23 cases with one false positive test (Figure 5 and Table 4). In the false positive case, Summit identified chromosome 1q gain and *MGMT* methylation in a patient who was determined to have infectious encephalitis upon clinician follow-up. *MGMT* methylation has been shown to occur in non-neoplastic conditions of the CNS, particularly inflammatory disease [18]. Of the 16 negative Summit tests, results for 14 samples confirmed the absence of primary CNS malignancy or therapeutic response and two tests were identified as false negatives (Table 5). One of the false negative specimens was determined to be astrocytoma upon biopsy with unique and uncharacteristic genomic variants that are not evaluated by Summit, and the other false negative sample was confirmed to be neoplastic but no molecular characterization data was available.

In the metastatic cohort, Summit informed management and/or treatment decisions in all 52 positive cases and informed the diagnosis (or lack) of LMD in 64 cases (Figure 5). All 31 negative cases in the metastatic cohort either confirmed absence of malignancy, absence of LMD, or therapeutic response in six negative cases with initial concern for leptomeningeal involvement, the absence of clinically significant variants in the CSF aided the provider in diagnosis of brain metastasis without LMD (Figure 5B,C). These six cases are considered false negative tests in assay performance calculations though Summit findings for these cases still resulted in CU outcomes.

The overall sensitivity of Summit in this single institution case series was observed to be 90% (75 true positive, 8 false negative) with a specificity of 97.5% (39 true negative, 1 false positive) performing better than initial validation studies [14]. Accuracy was demonstrated to be 93% (95% CI; range 86.56% to 96.60%) with a positive predictive value of the test was calculated to be 99% and a negative predictive value of 83% at 95% CI. Summit detected clinically significant variants in 76 of the 123 (62%) specimens and was negative in 47 (38%). This allowed Belay Summit testing to aid in clinical decision-making in 93% (114 of 123) of specimens, demonstrating strong CU (Figure 5D).

## 6. Discussion

The Belay tests demonstrated strong CU and influenced clinical decision-making in 93% (114 of 123) of the specimens from a single institution. In line with the previously published clinical validation [14], the results from this study reiterate that the Belay CSF liquid biopsy tests improve diagnostic accuracy, obviate biopsy, inform treatment decisions, and facilitate the diagnosis of LMD, particularly when traditional diagnostic methods are inadequate.

### 6.1. Summit Demonstrates Greater Sensitivity for Central Nervous System Malignancies Compared to Cerebrospinal Fluid Cytology

Though CSF cytology continues to play a role in the diagnosis of select CNS neoplasms such as LMD, its sensitivity remains suboptimal at about 50 to 60% on the first LP [19]. Summit identified tumor-informing variants in 56% of cases (59 of 106) with negative CSF cytology, and 100% of cases (4 of 4) with indeterminate CSF cytology, altering clinical management. This observation is consistent with previous studies demonstrating that CSF-based next generation sequencing (NGS) can detect malignant tDNA when cytology is negative or inconclusive, achieving sensitivity rates near 90% as demonstrated in our study [20]. Six cases with negative Summit results were found to have brain metastasis upon further diagnostic work-up, but the negative Summit findings helped inform the absence of LMD. As CSF-based molecular diagnostics become increasingly integrated into neuro-oncologic workflows, tests like Summit with low input DNA and higher sensitivity than CSF cytology mark a paradigm shift in the non-invasive management of CNS malignancies.

### 6.2. Summit Obviates Biopsy by Providing Molecular Characterization of Central Nervous System Tumors

CSF-based genomic analysis provides a safer alternative to surgical biopsies for CNS tumors particularly by mitigating procedural risks and enabling access to anatomically challenging regions [21]. Surgical biopsies carry risks such as non-diagnostic sampling (~10%), bleeding (1.7–4.7%), infection, stroke, seizures, and a mortality rate of up to 2% in serious cases [22]. These risks increase significantly in deep or critical brain regions, like the brainstem, and in frail or medically complicated individuals for whom biopsies may be too dangerous [21]. Results from this study show that Summit successfully detected informative molecular variants in patients with inaccessible lesions or poor surgical candidacy using CSF-tDNA analysis.

The diagnosis and treatment of primary CNS tumors leans heavily on molecular findings with increasing opportunities for targeted therapies [1,9]. In 15 of the 23 true positive primary CNS cases, Summit detected key biomarkers such as H3-3A K28M, a characteristic finding of diffuse midline glioma, that supported the provisional diagnosis and further characterized disease according to professional guidelines [1]. The detection of oncogenic hotspots variants in *IDH1* and *IDH2* aligned with parallel clinical findings and informed the use of IDH inhibitor vorasidenib in three cases. In another case, an *IDH1* variant was identified along with loss of chr9p21.3 (containing *CDKN2A* and *CDKN2B*) and a *TP53* loss-of-function variant [23]. These findings were concordant with the provisional diagnosis and informed further disease characterization as outlined by WHO diagnostic and prognostic criteria [1].

Moreover, growing evidence shows that genomic divergence exists between primary tumors, brain metastases, and LMD, reflecting the unique evolutionary pressures of the CNS microenvironment. Brain metastases and LMD often contain mutations not found in primary or extracranial tumors, highlighting branched clonal evolution and adaptation in the CNS, which is crucial for guiding targeted therapy [24]. In one of the cases, MRI findings initially raised concern for high-grade glioma (HGG) versus brain metastasis from known breast cancer. Summit results (detection of the PIK3CA Q546K variant with *ERBB2* amplification and high aneuploidy) were pivotal in confirming the lesion as a breast cancer metastasis, despite no radiographic or clinical evidence of systemic disease. This case highlights how CSF-tDNA analysis closes a critical gap in CNS malignancy diagnosis independent of cell morphology, making liquid biopsy not just an adjunct but necessary evolution in precision neuro-oncology [25].

### 6.3. Variant Allelic Frequencies and Aneuploidy Detected by Summit Inform Diagnosis and Monitoring of Leptomeningeal Disease

The standard use of CSF cytology in LMD diagnostic work-up yields 55% sensitivity upon the first examination of CSF. Sensitivity increases with repeated sampling, reaching up to 85% with three repetitions [2,26]. Frequent LPs to obtain a diagnosis are not ideal and the high-end sensitivity of CSF cytology still leaves room for improvement. Changes in VAF observed in CSF-tDNA longitudinal samples have been shown to correlate with clinical response and imaging findings, supporting the use of CSF liquid biopsy for dynamic disease monitoring [27]. This study demonstrates similar findings as Belay testing resulted in increased sensitivity for LMD compared to CSF cytology in the context of clinical presentation and neuroimaging. Analysis of VAF in metastatic cases in this study suggests a threshold of 5% VAF or above may distinguish LMD from parenchymal metastatic disease. This proposal is supported by 10 specific cases with VAFs >5%, 8 with initial clinical concern for LMD and 2 with suspected parenchymal metastasis. Parallel diagnostic work-up confirmed the presence of LMD in all 10 cases. Additionally, longitudinal Belay testing for two patients in the study illustrated how molecular findings, such as a loss of oncogenic variants or stable VAF, can mirror clinical presentation and support therapeutic response or lack thereof. While further research and larger cohorts are needed to assess utility of VAF tracking in LMD management, this single-institution experience serves as a promising foundation for optimizing this diagnostic tool.

Additionally, aneuploidy was detected in about a third of the positive metastatic cases, aiding in the diagnosis of LMD. High levels of aneuploidy are a known manifestation of chromosomal instability (CIN), a key driver of metastasis across cancer types [28]. However, in the review of available prior genomic profiling of plasma or primary tumors, most tests did not include analysis of chromosomal alterations. This observation may reflect a diagnostic gap that Summit addresses, especially in cases where metastasis is primarily driven by CIN or a single oncogenic driver is not well-characterized. The findings presented in this study align with recently published research in breast cancer that explores the use of aneuploidy in CSF as a biomarker for LMD [29]. Future longitudinal studies could further investigate whether a reduction in the number of chromosomal alterations, similar to a reduction in VAF, aligns with clinical signs of therapeutic response.

### 6.4. Summit Performance Is Not Affected by the Presence of Corticosteroids

Corticosteroids, commonly administered in patients with CNS tumors, are known to compromise CSF cytology and flow cytometry by reducing lymphoid cell counts [30]. About 32% of patients (24 of 76) with positive Summit results were taking a corticosteroid treatment at the time of sample collection, indicating that steroid use did not affect its performance. This observation aligns with previous reports showing that tDNA detection remains robust despite the immunosuppressive effects of corticosteroids, making it particularly valuable in real-world diagnostic settings where steroids are frequently introduced before a formal diagnosis [31].

### 6.5. Limitations and Future Directions

As CSF liquid biopsy is not yet standard practice, order indications for Belay testing are often cases with otherwise indeterminate diagnostic work-up or lesions unamenable to biopsy. This institutional experience is no exception and consequently the cases presented in this study have limited data available for strong concordance demonstrations of molecular findings, such as primary CNS tumor pathology. Additionally, the CSF cytology sensitivity of 12% in this cohort is considerably lower than what has been reported in the literature. This discrepancy is likely due to biased submission of specimens with inconclusive work-up, a workflow established prior to the conception of this retrospective study. Nevertheless, these findings demonstrate improved diagnostic yield with the use of CSF liquid biopsy in cases where CSF cytology fell short. Lastly, several of the authors of this study are employees of Belay Diagnostics and disclose considerable conflict of interest in reporting the CU of Summit. In an effort to mitigate this bias, CU outcome assessments were determined by a clinician of the collaborating institution who holds no stock or other affiliation with Belay Diagnostics.

While Summit results were used to inform care in the majority of cases in this study, a false positive rate of 3% (1/40) and false negative rate of 10% (8/83) were observed. As previously discussed, the molecular findings in the false positive case can also be seen in non-neoplastic etiologies and Summit results were considered in the context of a broader clinical picture, ultimately leaning on stronger evidence of infectious encephalitis. This example emphasizes that, like CSF cytology, Summit is not sufficient as a standalone diagnostic test and requires clinical correlation. False negative cases reflect Summit testing limitations such as variants present at low VAF or in genes/regions not included in the panel. Belay Diagnostics recently expanded the Summit panel with an increase in validated clinical sensitivity (96%) [32]. Summit 2.0 analyzes 488 genes for SNVs, MNVs, and Indels as well as copy-number variants in 63 genes, relevant fusions, tumor mutational burden, and microsatellite instability. Larger, multi-institutional studies are underway to assess the utility of the Summit 2.0 CSF-based liquid biopsy assay in larger patient populations from multiple centers.

## 7. Conclusions

This study presents the CU of the Belay CSF-tDNA Summit test as a tool to improve diagnostic accuracy for CNS malignancies, particularly in cases where CSF cytology results are negative or indeterminate and tissue diagnosis is infeasible. Notably, the Summit test influenced clinical decision-making in 93% (114 of 123) specimens by informing treatment and management decisions, demonstrating strong CU. Even with a refined panel, Belay testing was successfully integrated into one institution’s neuro-oncology clinical workflow and improved patient care. This experience serves as a roadmap for the implementation and optimal use of CSF-tDNA analysis to aid in the diagnosis and management of CNS malignancies.

## Figures and Tables

**Table 1:** Demographic characteristics of the single-institution cohort.

*Individual Patients (n = 120)*	Primary (*n* = 40; 33%)	Metastatic (*n* = 80; 67%)
***Sex assigned at birth***		
Male	20 (50%)	21 (26%)
Female	20 (50%)	59 (74%)
***Race***		
White	21 (53%)	40 (50%)
Hispanic/Latino	6 (15%)	8 (10%)
Black or African American	2 (5%)	11 (14%)
Asian or Pacific Islander	2 (5%)	8 (10%)
Middle Eastern	1 (3%)	1 (1%)
Not Provided	8 (20%)	12 (15%)
***Age range (years)***		
1–20	3 (8%)	0
21–50	15 (38%)	19 (24%)
51–65	8 (20%)	32 (40%)
>65	14 (35%)	29 (36%)

**Table 2:** Test order considerations for 123 cerebrospinal fluid (CSF) specimens submitted for Summit testing.

*CSF Specimen Collection Site*	
Lumbar puncture	114 (93%)
Ommaya reservoir	9 (7%)
***Location of disease***	
Brain	94 (76%)
Spine	8 (7%)
Both	21 (17%)
***Symptoms at time of order***	
None	27 (22%)
Cranial nerve deficit	37 (30%)
Headaches	35 (28%)
Motor difficulty	27 (22%)
Altered mental status	16 (13%)
Back pain	9 (7%)
Sensory issues	6 (5%)
Seizures	4 (3%)
Dizzy/balance issues	2 (2%)

**Table 3:** Variants detected by Summit (red) in specimens with suspected metastatic disease in concordance with results from previous tumor or liquid biopsy genomic profiling results (only variants included in the Summit panel are listed).

Study ID	Tissue of Origin	Chromosomal Arm-Level Variants Detected by Summit	Gene-Level Variants Detected by Summit	Variants Detected in Prior Blood/Plasma Genomic Reports	Variants Detected in Prior Primary or Metastatic Tumor Genomic/IHC Reports
26	Breast	None	CTNNB1 S33Y TP53 R248P	N/A	*Primary* HRAS Q61L PIK3CA H1047R
27	Breast	chr1q gain, chr2q gain, chr3q gain, chr4q gain, chr5p gain, chr6q loss, chr8p loss, chr8q gain, chr9p loss, chr9q gain, chr12q gain, chr13q gain, chr17q gain (*ERBB2* amp), chr20p loss, chr20q gain, chr21q gain	PIK3CA 1047R TP53 R280T TP53 D259fs	*ERBB2* amp PIK3CA H1047R TP53 D259fs	N/A
22	Breast	chr1p loss, chr1q gain, chr2p gain, chr2q gain, chr3p gain, chr3q gain, chr4p loss, chr4q loss, chr5p gain, chr5q gain, chr6p gain, chr6q gain, chr7p gain, chr7q gain, chr8p gain, chr8q gain, chr9p gain, chr9q gain, chr10p gain, chr10q gain, chr11p loss, chr11q gain, chr12p gain, chr12q gain, chr13q loss, chr14q gain, chr15q loss, chr16p gain, chr16q loss, chr17p loss, chr18p loss, chr19p gain, chr20p gain, chr20q gain, chr21q loss, chr22q loss	CDH1 Q23 * PIK3CA H1047L PTEN P246fs	CDH1 Q23 * FGFR2 Y376C KRAS Q61K PIK3CA H1047L PTEN 246fs SMAD4 S154 *	N/A
40	Lung	None	EGFR T790M	EGFR T790M EGFR L858R TP53 L130V	N/A
14	Skin	None	None	N/A	*Brain Metastatic Site* BRAF V600K
13	Breast	None	AKT1 E17K	AKT1 E17K CDH1 A575fs TP53 R181H	N/A
1	Lung	None	None	EGFR L858R PIK3CA E545K TP53 R213 *	N/A
3	Breast	None	None	GATA3 T419fs TP53 K132R	N/A
9	Lung	chr1p gain, chr5p gain, chr6p loss, chr6q loss, chr7p gain, chr7q gain, chr9p loss, chr11p gain, chr15q gain, chr17q gain, chr19p loss, chr19q loss, chr20p gain, chr20q gain, chr21q loss, chr22q loss	APC C1502fs EGFR E746_A750del EGFR C797S PTEN R130Q	EGFR mutant NOS	N/A
4	Lung	chr2p loss, chr4q gain, chr5p loss, chr7q loss, chr10q gain, chr11q gain, chr12q loss, chr13q gain, chr14q loss, chr16p loss, chr17p loss, chr19q loss	None	N/A	*Primary* EGFR L858R
19	Breast	None	None	N/A	*Primary* TP53 I195T
21	Breast	None	TP53 S94 *	*ERBB2* amp TP53 S94 *	N/A
10	Lung	None	TP53 R181C TP53 A138P	EGFR L858R TP53 R181C TP53 Q375fs*80	*Bone Metastatic Site* EGFR L858R *ERBB2* amp TP53 Q375fs*80 TP53 R181C
29	Lung	chr3q gain, chr8q gain, chr14q gain, chr12p gain	TP53 A138fs TP53 G245S TP53 R273H	TP53 C242fs*5 TP53 G245S TP53 R273H	N/A
73	Breast	chr6p loss, chr13q loss	None	PIK3CA H1047R	N/A
77	Breast	None	TP53 S215R TP53 V216M TP53 G266R	TP53 V216M	N/A
96	Lung	None	TP53 L257Q	N/A	*Lymph Node Metastatic Site* TP53 L257Q

Chromosome, chr; not otherwise specified, NOS; amplification, amp. * indicates truncating variant.

**Table 4:** Clinically actionable alterations detected by Summit in cerebrospinal fluid (CSF) samples with suspected primary and metastatic central nervous system (CNS) disease.

Study ID	Suspected Tissue of Origin	CSF Cytology Results	Clinically Significant Variants Detected			Summit Clinical Utility
			Gene-Level Variant	Chromosomal Arm-Level Aneuploidy	*MGMT* Promoter Methylation	
51	CNS	Negative	Yes	No	Not ordered	Inform Treatment
52	CNS	Indeterminate	No	Yes	Not ordered	Inform Treatment
53	CNS	Indeterminate	Yes	Yes	Methylated	Inform Diagnosis and Treatment
54	CNS	Negative	No	Yes	Unmethylated	Inform Treatment
55	CNS	Negative	Yes	Yes	Unmethylated	Inform Treatment
56	CNS	Negative	Yes	No	Not ordered	Inform Treatment
57	CNS	Negative	Yes	No	Unmethylated	Inform Treatment
58	CNS	Negative	Yes	No	Unmethylated	Inform Management
59	CNS	Negative	Yes	Yes	Indeterminate	Inform Treatment
60	CNS	Negative	Yes	Yes	Indeterminate	Inform Diagnosis and Treatment
61	CNS	Negative	Yes	Yes	Not ordered	Inform Diagnosis and Treatment
62	CNS	Negative	Yes	Yes	Unmethylated	Inform Management
65	CNS	Negative	No	Yes	Methylated	False Positive
69	Lymphatic	Negative	Yes	Yes	Not ordered	Inform Diagnosis
80	Lymphatic	Negative	Yes	No	Unmethylated	Inform Diagnosis and Treatment
88	Lymphatic	Negative	Yes	No	Not ordered	Inform Diagnosis
94	Lymphatic	Negative	Yes	No	Unmethylated	Inform Diagnosis
98	CNS	Negative	Yes	No	Not ordered	Inform Diagnosis and Treatment
100	Lymphatic	Negative	Yes	No	Not ordered	Inform Diagnosis
108	CNS	Negative	Yes	No	Unmethylated	Inform Management
110	Lymphatic	Negative	Yes	No	Not ordered	Inform Diagnosis
117	CNS	Negative	Yes	No	Unmethylated	Inform Diagnosis
118	CNS	Negative	No	Yes	Not ordered	Inform Diagnosis
119	Lymphatic	Not Performed	Yes	No	Not ordered	Inform Diagnosis
20	CNS	Negative	Yes	No	Methylated	Inform Management
8	Breast	Negative	Yes	No	Not ordered	Inform Treatment
13	Breast	Negative	Yes	No	Not ordered	Inform Treatment
17	Breast	Negative	Yes	No	Not ordered	Inform Treatment
22	Breast	Positive	Yes	Yes	Not ordered	Inform Treatment
23 *	Breast	Positive	Yes	No	Not ordered	Inform Treatment
24	Breast	Positive	Yes	Yes	Not ordered	Inform Treatment
25	Breast	Positive	Yes	Yes	Unmethylated	Inform Treatment
26	Breast	Negative	Yes	No	Not ordered	Inform Treatment
27	Breast	Negative	Yes	Yes	Not ordered	Inform Treatment
33	Breast	Negative	Yes	Yes	Not ordered	Inform Treatment
37	Breast	Negative	Yes	Yes	Not ordered	Inform Treatment
38	Breast	Negative	Yes	Yes	Unmethylated	Inform Treatment
39	Kidney	Negative	Yes	No	Not ordered	Inform Treatment
4	Lung	Negative	No	yes	Not ordered	Inform Treatment
9	Lung	Negative	Yes	Yes	Unmethylated	Inform Treatment
28	Lung	Negative	Yes	Yes	Unmethylated	Inform Management
29	Lung	Negative	Yes	Yes	Unmethylated	Inform Treatment
40	Lung	Negative	Yes	No	Not ordered	Inform Treatment
30	Ovary	Negative	Yes	No	Not ordered	Inform Treatment
11	Pancreas	Negative	Yes	Yes	Unmethylated	Inform Treatment
21	Breast	Negative	Yes	No	Unmethylated	Inform Treatment
10	Lung	Negative	Yes	No	Not ordered	Inform Treatment
63	Lymphatic	Negative	No	Yes	Methylated	Inform Treatment
64	CNS	Negative	Yes	No	Unmethylated	Inform Treatment
66 *	Breast	Indeterminate	Yes	No	Unmethylated	Inform Treatment
67	Lung	Positive	Yes	Yes	Unmethylated	Inform Treatment
68	Blood	negative	Yes	No	Not ordered	Inform Diagnosis
70	Lung	Negative	Yes	Yes	Unmethylated	Inform Treatment
72	Kidney	Negative	No	Yes	Unmethylated	Inform Diagnosis
76	Breast	Not performed	Yes	Yes	Not ordered	Inform Diagnosis
77	Breast	Negative	Yes	No	Not ordered	Inform Treatment
81	Breast	Positive	Yes	Yes	Not ordered	Inform Diagnosis and Management
83	Lung	Positive	Yes	No	Not ordered	Inform Treatment
84	Blood	Negative	Yes	No	Not ordered	Inform Management
89	Lung	Negative	Yes	Yes	Not ordered	Inform Management
90	Lymphatic	Positive	Yes	No	Not ordered	Inform Diagnosis and Treatment
91	Pancreatic	Negative	Yes	No	Not ordered	Inform Diagnosis and Treatment
93	Prostate	Not performed	Yes	Yes	Not ordered	Inform Diagnosis and Treatment
95	Gastric	Indeterminate	Yes	Yes	Not ordered	Inform Diagnosis and Management
96	Lung	Positive	Yes	No	Not ordered	Inform Diagnosis and Treatment
99	Lymphatic	Negative	Yes	No	Not ordered	Inform Diagnosis
105	Blood	Negative	No	Yes	Unmethylated	Inform Diagnosis
106	Breast	Negative	Yes	No	Not ordered	Inform Management
107	Lymphatic	Negative	No	Yes	Not ordered	Inform Diagnosis
111	Colon	Positive	Yes	Yes	Unmethylated	Inform Diagnosis
112	Blood	Negative	Yes	No	Not ordered	Inform Diagnosis
114	Lymphatic	Negative	No	Yes	Not ordered	Inform Diagnosis
115	Anal	Negative	Yes	No	Not ordered	Inform Diagnosis
116	Breast	Negative	Yes	Yes	Not ordered	Inform Diagnosis and Treatment
122	Lymphatic	Negative	Yes	Yes	Not ordered	Inform Diagnosis
123	Lung	Negative	Yes	No	Unmethylated	Inform Management

* Specimens from the same patient; cerebrospinal fluid.

**Table 5:** Follow-up of Summit-negative cases post testing to determine specificity and negative predictive value.

Study ID	Suspected Tissue of Origin	CSF Cytology Results	Summit Concordance with Clinical Information	Clinical Follow-Up Post-Belay Testing	Summit Clinical Utility
**Primary CNS Cases (*n* = 16)**					
41	CNS	Negative	True Negative	CSF was sent post-biopsy and/or treatment and Summit testing confirmed the expected negative result.	Confirmed therapeutic response
42	CNS	Negative			
43	CNS	Negative			
46	CNS	Negative			
48	CNS	Negative			
50	CNS	Negative			
44	CNS	Negative		Creutzfeldt-Jakob Disease	Confirmed absence of malignancy
45	CNS	Negative		Demyelinating disease	
47	CNS	Negative		Hemangiopericytoma, no disease in CSF	
49	CNS	Negative		Demyelinating disease, confirmed to be negative on biopsy	
78	CNS	Negative		Non-malignancy, observation	
104	CNS	Negative		Observation	
120	CNS	Negative		Diagnosis	
121	CNS	Negative		Diagnosis	
103	CNS	Negative	False Negative	Unmethylated high-grade astrocytoma with unique genomic profiling, not consistent with glioblastoma	False Negative
97	CNS	Negative		Surgery showed presence of neoplasm, no molecular profiling results were available	
**Metastatic CNS Cases (*n* = 31)**					
3	Breast	Negative	True negative for LMD, False negative for cancer	No LMD, dural invasion of right parietal bone	Confirmed absence of LMD
32	Breast	Negative		No LMD, low grade dural lesion	
6	Breast	Negative		No LMD, pachymeningeal disease, likely dural based metastasis	
51	Breast	Negative		No LMD, radiation for brain metastasis	
21	CNS	Negative		No LMD, radiation for brain metastasis	
82	Thyroid	Negative		No LMD, radiation for brain metastasis	
12	Breast	Negative	True Negative	Recent craniospinal irradiation prior to Belay testing	Confirmed therapeutic response
19	Breast	Negative		Recent therapy course	
31 *	Ovary	Negative		Therapeutic response (serial testing)	
86	Breast	Negative		Continue therapy	
101 **	Lung	Negative		Observation	
34	Pancreas	Negative		No intracranial disease	Confirmed absence of malignancy
15	Prostate	Negative		Inflammatory disease from immunotherapy	
18	Prostate	Negative		Transient inflammation	
14	Skin	Negative		Radiation necrosis	
36	Bone Marrow	Negative		No malignancy in CNS	
16	Colon	Negative		Inflammatory, no malignancy	
7	Kidney	Negative		Multiple sclerosis	
1 **	Lung	Negative		No LMD, low grade brain metastasis, follow-up CSF cytology negative	
2	Lung	Negative		No neoplasm seen on follow-up	
35	Lymphatic	Negative		Unclear presentation	
5	Breast	Negative		Over shunting	
71	Breast	Negative		No malignant	
74	Lymphatic	Negative		Demyelinating disease	
75	CNS	Negative		Diagnosis	
79	Prostate	Negative		Observation	
82	Cervical	Negative		Observation	
87	Colon	Negative		Observation	
92	Lymphatic	Negative		Systemic therapy with no target of CNS	
109	Lymphatic	Negative		Diagnosis	
113	Lymphatic	Negative		Diagnosis	

* Repeat specimen with previous positive testing; ** Specimens from the same patient; cerebrospinal fluid, CSF; central nervous system, CNS; leptomeningeal disease, LMD.

## Data Availability

Data will be made available upon reasonable request.
